# Rhythmic Walking Exercise as a Low-Intensity Strategy to Enhance Health and Preserve Kidney Function in Individuals with CKD Stages 2–3

**DOI:** 10.3390/life15111735

**Published:** 2025-11-12

**Authors:** Pattraphon Suvannarot, Thapanee Roengrit, Promtpong Anuchitchanchai, Piyapong Prasertsri

**Affiliations:** 1 Department of Physical Therapy, Chamni Hospital, Buriram 31110, Thailand; pummywnk25@gmail.com; 2 Institute of Medicine, Suranaree University of Technology, Nakhon Ratchasima 30000, Thailand; thapanee.ro@sut.ac.th; 3 Department of Orthopedics, Faculty of Medicine, Burapha University, Chonburi 20131, Thailand; promtpong@yahoo.co.th; 4 Faculty of Allied Health Sciences, Burapha University, Chonburi 20131, Thailand

**Keywords:** chronic kidney disease, rhythmic walking, oxidative stress, blood pressure, superoxide dismutase, malondialdehyde, exercise intervention, renal function

## Abstract

Physical inactivity contributes to oxidative stress, hypertension, and progressive kidney dysfunction in individuals with chronic kidney disease (CKD). Rhythmic walking, a low-intensity and easily implemented form of exercise, may offer renal and cardiovascular benefits. This randomized controlled trial examined the effects of rhythmic walking in adults with CKD stages 2–3. Sixty-four participants (mean age: 67.8 ± 7.5 years) were randomly assigned to a control group (*n* = 33; standard care) or a walking group (*n* = 31; 30 min/session, three times weekly for 12 weeks at 60 steps/min). Primary outcomes included kidney function, oxidative stress, and blood pressure. After 12 weeks, no significant within-group changes were observed in kidney function, though the walking group showed trends toward higher estimated glomerular filtration rate (*p* = 0.071) and estimated creatinine clearance (*p* = 0.089). Between-group analysis revealed significantly higher superoxide dismutase activity (*p* = 0.028) and lower malondialdehyde levels (*p* = 0.037) in the walking group. Both groups exhibited blood pressure reductions, with greater decreases in pulse rate (*p* = 0.016) and rate–pressure product (*p* = 0.039) in the walking group. A 12-week rhythmic walking program improved oxidative stress and BP profiles and may help slow renal decline in CKD.

## 1. Introduction

Chronic kidney disease (CKD) is characterized by structural and functional kidney abnormalities arising from diverse causes. Its global prevalence is ~13% (11–15% across countries) and continues to increase annually [1]. Individuals with CKD face a 40-fold higher risk of mortality compared with the general population [2], making it a major contributor to global morbidity and mortality [1].

Early-stage CKD (stages 1–2) is typically asymptomatic due to minimal damage, with symptoms often emerging at stage 3 when functional decline prompts medical care. By this point, opportunities for early intervention are frequently missed, accelerating progression toward stage 5 or end-stage renal disease, where dialysis and kidney transplantation are the only treatment options. This progression imposes substantial financial and social burdens. Therefore, prevention and management during stages 1–4 are critical to slowing disease progression and reducing its overall impact [2,3].

Physical activity is a key component of CKD management, complementing pharmacological treatment, dietary control, and antihypertensive therapy. Regular exercise improves overall health, enhances functional capacity, prevents complications, lowers cardiovascular risk, and reduces mortality in CKD patients [4,5,6,7,8]. Physiologically, exercise enhances renal activity, modulates blood flow and electrolyte balance, and improves systemic circulation [9]. Beyond these effects, it also improves health-related quality of life and provides psychological benefits, including reduced anxiety, stress, and depression [10].

Recent evidence suggests that patients with nondialysis CKD (stages 1–4) should interrupt prolonged sedentary periods with light physical activity, as even brief bouts of standing or movement confer measurable benefits. Findings from the general population are likely applicable to CKD patients [11]. Supporting this, our previous trial in older adults with hypertension demonstrated that both continuous and intermittent rhythmic walking, performed for 30 min per day over 12 weeks, enhanced antioxidant capacity, reduced oxidative stress, and improved blood pressure, metabolic profiles, and cardiac autonomic function, while increasing daily step counts by ~1800 steps [12]. Moreover, walking for 30 min per day on most days of the week provides significant health benefits in patients with kidney disease [10].

Despite these benefits, many individuals with nondialysis CKD remain sedentary, a behavior strongly linked to adverse health outcomes and functional decline. Observational studies show that patients with CKD engage in physical activity on only ~9 days per month, and 45% of those with end-stage kidney disease report no exercise at all [13]. To address this gap, the present study implemented a rhythmic walking program in individuals with CKD stages 2–3, a critical phase where timely intervention may delay further functional decline. Specifically, we tested the hypothesis that rhythmic walking would improve kidney function, oxidative stress, and blood pressure, with additional benefits for metabolic profile, body composition, and physical fitness.

## 2. Materials and Methods

### 2.1. Study Design and Sample Size

This randomized controlled trial was conducted between October 2024 and May 2025. The study population comprised patients with CKD stages 2–3 residing in Buriram Province. The sample size was determined based on a previous study that evaluated a 12-week, 30 min home-based walking exercise program in patients with CKD stages 3–4 [14]. In that study, the mean post-intervention glomerular filtration rate (GFR) was 31.9 ± 13.7 mL/min/1.73 m^2^ in the experimental group and 23.9 ± 12.2 mL/min/1.73 m^2^ in the control group, corresponding to an effect size of 0.61. Assuming a two-tailed alpha of 0.05 and a power of 0.80, the required sample size was calculated using statistical software [15], resulting in 34 participants per group. To account for a potential 10% dropout rate, the sample size was increased to 37 participants per group, yielding a total of 74 participants for the study.

### 2.2. Ethical Approval and Trial Registration

The study was conducted in accordance with the principles of the Declaration of Helsinki and approved by the Human Research Ethics Committee of the Buriram Provincial Public Health Office (approval no. BRO 2024-074; approval date: 16 July 2024). All participants provided written informed consent prior to participation. The trial was retrospectively registered at ClinicalTrials.gov (Identifier: NCT07059559; registration date: 1 July 2025).

### 2.3. Participant Recruitment and Screening

The researcher conducted participant recruitment and screening at the Out-Patient Department of Chamni Hospital, Chamni District, Buriram Province. The inclusion criteria were as follows: (a) male or female patients aged 35–80 years and (b) diagnosed with CKD stage 2–3, defined by an estimated GFR (eGFR) between 30 and 89 mL/min/1.73 m^2^ consistently for at least three months. The exclusion criteria were: (a) presence of musculoskeletal disorders that interfere with walking, such as osteoarthritis; (b) regular engagement in exercise exceeding three sessions per week or 150 min per week; (c) habitual use of medications or supplements containing antioxidants; (d) diagnosis of schizophrenia or other psychiatric conditions; and (e) inability to communicate in the Thai language.

### 2.4. Participant Randomization and Allocation

All participants who provided written informed consent and met the eligibility criteria during screening were randomly allocated to either the control or walking exercise group based on the sequence of their enrollment. To ensure simplicity and feasibility in clinical implementation, randomization was performed according to the order of enrollment, following the principle of simple randomization, in which each participant had an equal and independent chance of assignment. Participants with odd enrollment numbers (e.g., 1, 3, 5, …) were assigned to the control group (standard hospital care), while those with even numbers (e.g., 2, 4, 6, …) were assigned to the walking exercise group.

To maintain allocation concealment, all participants were recruited on a single day during their routine outpatient visits. None were aware of their enrollment sequence, and group assignments were disclosed only after screening was completed. This study was non-blinded: participants were aware of their group allocation. The principal researcher (P.S.) was responsible for participant recruitment, screening, randomization, allocation, data collection, and outcome assessments, while data analysis was independently performed by P.P. and T.R.

### 2.5. Intervention Procedures

The walking exercise program consisted of continuous walking for 30 min per day, three days per week, over a 12-week period. Each session included a 5 min warm-up (gentle stretching and slow walking), 30 min of rhythmic walking at the target pace, and a 5 min cool-down period with light movements and breathing exercises. The walking cadence was standardized at approximately 60 steps per minute (equivalent to one step per second).

Before starting the intervention, participants received individualized instruction and a practical demonstration from the researcher to ensure proper walking technique, posture, and pacing. Each participant practiced under supervision until they could comfortably maintain the target cadence. The walking sessions were then performed independently at home. To facilitate adherence, participants used a mobile pacing application that provided auditory cues to help maintain the prescribed rhythm.

**Figure 1:**
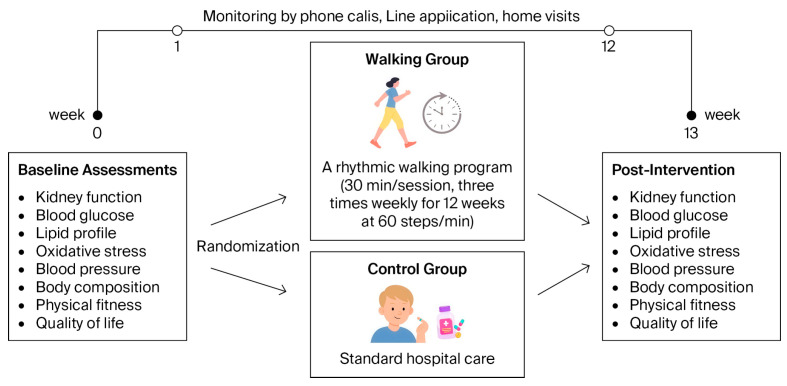
Intervention procedures of the study, illustrating the timeline of baseline assessments, randomization, intervention, and posttest measurements.

Exercise adherence—classified as high (≥80%) or low (<80%)—and safety were monitored throughout the 12-week program. Participants documented each exercise session in a provided logbook, which was verified weekly by the researcher (P.S.) through phone calls or the Line application. Any symptoms of discomfort, pain, or adverse events were documented and promptly followed up. Both groups were instructed to maintain their usual daily activities during the 12-week study period (Figure 1).

### 2.6. Determination of Kidney Function

In addition to creatinine and uric acid levels, kidney function was determined by eGFR [16] and estimated creatinine clearance (eCrCl) [17] using the following formulas:

eGFR (mL/min/1.73 m^2^) = 175 × [serum creatinine (mg/dL)/88.4] − 1.154 × (age) − 0.203 × (0.742 if female)

eCrCl (mL/min) = [(140 − age) × body weight × (0.85 if female)]/[72 × serum creatinine (mg/dL)]

### 2.7. Laboratory Tests

Approximately 10 mL of venous blood was collected from the antecubital vein by a certified medical technician at Chamni Hospital. The samples were divided into sodium fluoride/potassium oxalate, clotted blood, and EDTA tubes for the measurement of blood glucose (BG), lipid profile (total cholesterol [TC], high-density lipoprotein cholesterol [HDLC], low-density lipoprotein cholesterol [LDLC], and triglycerides [TG]), uric acid, and creatinine. All analyses were performed and standardized by the Department of Medical Technology, Chamni Hospital, and the RIA Laboratory Co., Ltd., Nakhon Ratchasima, Thailand. In addition, the ratios of TC/HDLC, LDLC/HDLC, and TG/HDLC were subsequently calculated.

Biomarkers of oxidative stress included superoxide dismutase (SOD) activity and malondialdehyde (MDA). Level of serum SOD activity (% inhibition), an indicator of antioxidant defense, was measured using a colorimetric method with the SOD Assay Kit-WST (Dojindo Laboratories, Kumamoto, Japan) following the manufacturer’s instructions. Level of plasma MDA (µM), a biomarker of lipid peroxidation, was measured using a spectrophotometric method, as previously described [18].

### 2.8. Blood Pressure Measurement

Systolic and diastolic blood pressure (SBP and DBP) and pulse rate (PR) were measured using an automatic BP monitor (HEM-7121, Omron Healthcare Co., Ltd., Kyoto, Japan), as previously described [19]. Mean arterial pressure (MAP), pulse pressure (PP), and rate–pressure product (RPP) were subsequently calculated as follows [20]:

PP (mmHg) = SBP – DBP; MAP (mmHg) = DBP + 1/3 PP; RPP (mmHgbpm) = SBP × PR

### 2.9. Anthropometry, Body Composition, and Fat Distribution Measurements

Height and weight were measured using standard equipment (Health O meter Pro Series, McCook, IL, USA), and BMI were calculated using the formula:

BMI (kg/m^2^) = weight (kg)/height^2^ (m)

Body composition parameters, including muscle mass, fat mass, bone mass, visceral fat level (VFL), and basal metabolic rate (BMR), were assessed using a bioelectrical impedance analyzer (Tanita UM-076, Tokyo, Japan). In addition, body fat distribution was determined by measuring waist and hip circumferences with a standard measuring tape, following the procedure described by Prasertsri et al. [19]. The waist-to-hip ratio (WHR) was subsequently calculated.

### 2.10. Physical Fitness Tests

Handgrip strength, an indicator of overall musculoskeletal strength capacity, was assessed as described previously [21]. Participants were seated with the shoulder adducted and neutrally rotated, the elbow flexed at 90°, and the forearm and wrist in a neutral position. They were then instructed to grip a hand dynamometer (T.K.K. 5001, Grip-A, Tokyo, Japan) with maximal effort. Three trials were performed for each hand, and the highest value was recorded for analysis. Handgrip strength was evaluated for both the dominant and non-dominant hands.

The 60 s sit-to-stand (60STS) test was performed to assess lower limb muscle strength and exercise capacity. Participants sat on a standard armless chair (height: 46 cm) with their hands placed on their hips and were allowed one to two practice attempts before commencing the test. They were instructed to perform as many full sit-to-stand repetitions as possible at a self-selected pace within 60 s. The number of repetitions was counted from the initial sitting position to the final completed stand (i.e., when the legs were fully extended before time elapsed) [22].

### 2.11. Quality of Life Assessment

Quality of life (QOL) was assessed using the WHOQOL-BREF-THAI questionnaire, which demonstrated good internal consistency (Cronbach’s alpha = 0.84) and acceptable validity (0.65 compared with the WHOQOL-100) [23]. The questionnaire consists of 26 items: 2 items assess overall QOL and general health, and the remaining items are grouped into four domains—physical health (7 items), psychological health (6 items), social relationships (3 items), and environment (8 items). Each item is rated on a 5-point Likert scale ranging from 1 (“not at all”) to 5 (“completely”). The total score categorizes QOL as good (96–130), fair (61–95), or poor (26–60).

### 2.12. Data Analyses

Descriptive statistics were expressed as frequencies, percentages, means, and standard deviations (SD). The normality of data distribution and homogeneity of variances were assessed using the Shapiro–Wilk and Levene’s tests, respectively. Baseline differences between groups were analyzed using independent *t*-tests. Post-intervention differences within and between groups were evaluated using repeated-measures analysis of variance (ANOVA) with Bonferroni post hoc correction. Where appropriate, paired *t*-tests were conducted to verify within-group changes. In cases where baseline differences were observed, analysis of covariance (ANCOVA) with Bonferroni adjustment was applied. For all primary outcomes, 95% confidence intervals (CIs), effect sizes (partial eta-squared, η^2^), and *p*-values were reported. Statistical analyses were performed using SPSS Statistics version 25 (IBM Corp., Armonk, NY, USA), with the level of significance set at *p* < 0.05.

## 3. Results

**Figure 2:**
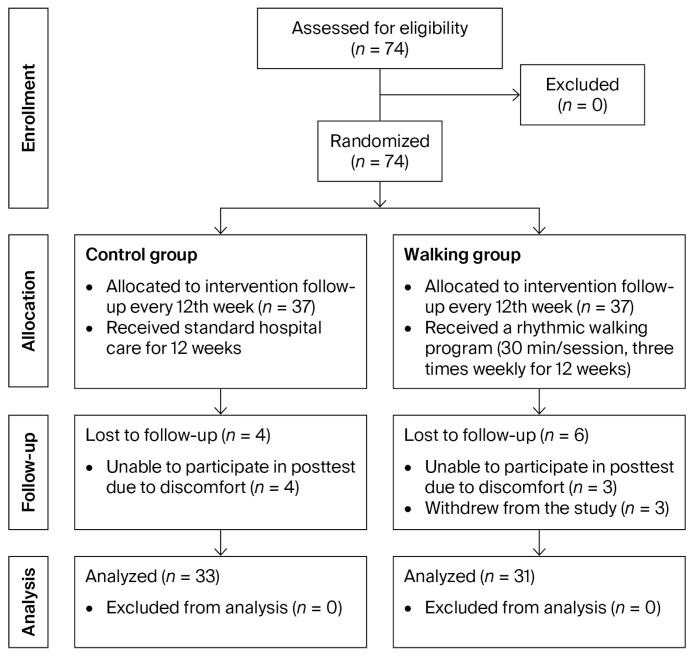
CONSORT flow diagram of participant enrollment, allocation, follow-up, and analysis.

Participants were recruited between October and December 2024, and follow-up assessments were conducted from April to May 2025, coinciding with the final participant visits. A total of 74 participants were enrolled and randomized equally into two groups (37 participants per group). Of these, 10 participants did not complete the study due to withdrawal from participation (*n* = 3) or unavailability for the post-intervention assessment (*n* = 7). Consequently, data from 64 participants were included in the final analysis (Figure 2).

The control group included 33 participants (14 males [42%] and 19 females [58%]) with a mean age of 69.03 ± 6.96 years and a mean height of 155.00 ± 11.23 cm. The walking group consisted of 31 participants (9 males [29%] and 22 females [71%]) with a mean age of 66.45 ± 7.91 years and a mean height of 152.84 ± 9.55 cm. There were no statistically significant differences between the two groups in mean age (*p* = 0.170) or height (*p* = 0.412). Exercise adherence in the walking group was considered high, with an average compliance rate of 81%.

### 3.1. Baseline Clinical Characteristics of Participants

In the control group, 26 participants (78.8%) were classified as CKD stage 2 and 7 (21.2%) as stage 3. Similarly, in the walking group, 20 participants (64.5%) were in stage 2 and 11 (35.5%) in stage 3. The most common underlying diseases were hypertension (93.94% vs. 93.55%), dyslipidemia (39.39% vs. 58.06%), type 2 diabetes (21.21% vs. 25.81%), old transient ischemic attack (12.12% vs. 12.90%), gouty arthritis (6.06% vs. 12.90%), and thyroid disease (6.06% vs. 0%). Current cigarette smoking (18.18% vs. 3.23%) and alcohol consumption (24.24% vs. 19.35%) were also reported in both groups (Table 1).

### 3.2. Kidney Function and Biochemical Parameters

After the 12-week intervention, levels of creatinine (*p* = 0.447 and *p* = 0.778), eGFR (*p* = 0.726 and *p* = 0.071), and eCrCl (*p* = 0.537 and *p* = 0.089) showed no significant changes in either the control or walking group. Notably, there was a trend toward increased eGFR and eCrCl levels in the walking group. Between-group comparisons revealed no significant differences in any of these parameters (creatinine: 95% CI: −0.07 to 0.09, partial η^2^ = 0.002, *p* = 0.744; eGFR: 95% CI: −7.68 to 1.62, partial η^2^ = 0.027, *p* = 0.197; eCrCl: 95% CI: −6.52 to 2.15, partial η^2^ = 0.016, *p* = 0.318) (Table 2).

Further biochemical analyses showed that uric acid (*p* = 0.552), BG (*p* = 0.213), TC (*p* = 0.692), HDLC (*p* = 0.213), LDLC (*p* = 0.790), TG (*p* = 0.926), TC/HDLC ratio (*p* = 0.940), LDLC/HDLC ratio (*p* = 0.729), and TG/HDLC ratio (*p* = 0.555) did not significantly change in the control group. Similar findings were observed in the walking group for uric acid (*p* = 0.657), BG (*p* = 0.181), HDLC (*p* = 0.101), and LDLC/HDLC ratio (*p* = 0.095), except for TC (*p* = 0.003), LDLC (*p* = 0.021), TG (*p* = 0.022), and TG/HDLC ratio (*p* = 0.048), which significantly decreased after the intervention, with a trend toward a lower TC/HDLC ratio (*p* = 0.057) (Table 2).

**Figure 3:**
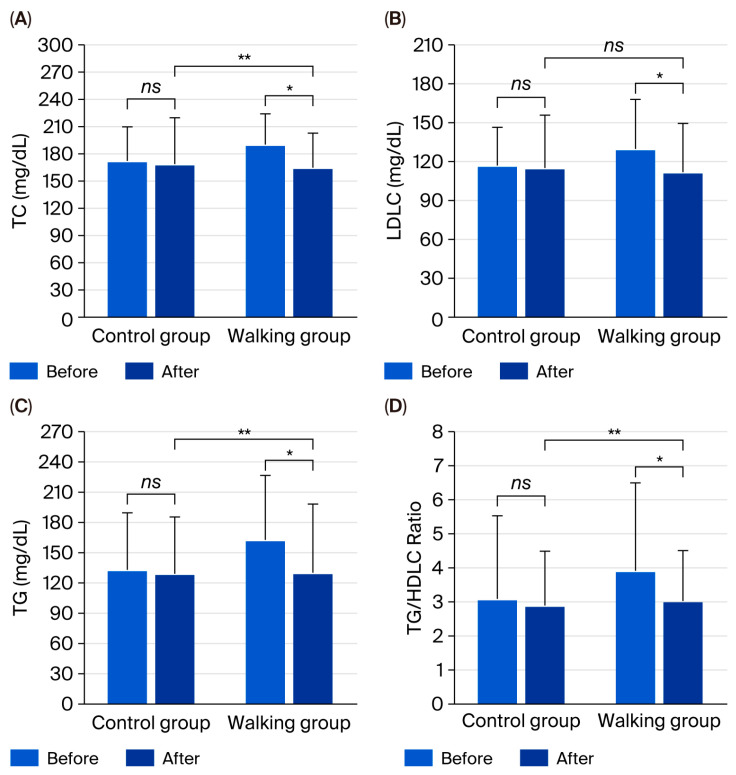
Total cholesterol (TC) (**A**), low-density lipoprotein cholesterol (LDLC) (**B**), triglycerides (TG) (**C**), and TG/HDLC ratio (**D**) in control and walking groups at baseline and after 12 weeks. Data are mean ± SD. * *p* < 0.05 vs. before intervention; ** *p* < 0.05 vs. control group; *ns* = not significant (*p* > 0.05).

Between-group comparisons revealed significantly lower levels of TC (95% CI = −35.76 to −1.85, partial η^2^ = 0.076, *p* = 0.030), TG (95% CI = −53.55 to −3.93, partial η^2^ = 0.083, *p* = 0.024), and TG/HDLC ratio (95% CI = −1.86 to −0.09, partial η^2^ = 0.076, *p* = 0.032) in the walking group compared with the control group (Figure 3). No significant between-group differences were observed in uric acid (95% CI = −0.47 to 0.41, partial η^2^ = 0.000, *p* = 0.896), BG (95% CI = −16.73 to 10.25, partial η^2^ = 0.004, *p* = 0.633), HDLC (95% CI = −6.67 to 3.23, partial η^2^ = 0.008, *p* = 0.490), or LDLC/HDLC ratio (95% CI = −0.84 to 0.15, partial η^2^ = 0.031, *p* = 0.170). However, there was a tendency toward lower LDLC (95% CI = −29.67 to 2.01, partial η^2^ = 0.048, *p* = 0.086) and TC/HDLC ratio (95% CI = −1.04 to 0.04, partial η^2^ = 0.053, *p* = 0.071) in the walking group.

### 3.3. Oxidative Stress

**Figure 4:**
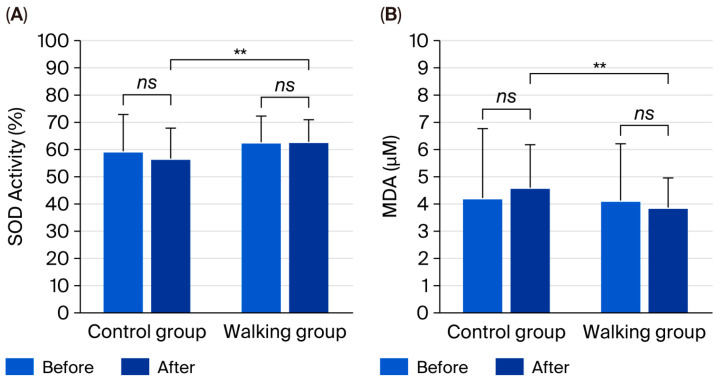
Superoxide dismutase (SOD) activity (**A**) and malondialdehyde (MDA) levels (**B**) in control and walking groups at baseline and after 12 weeks. Data are mean ± SD. ** *p* < 0.05 vs. control group; *ns* = not significant (*p* > 0.05).

There were no significant within-group changes in SOD activity (*p* = 0.900 and *p* = 0.352) or MDA (*p* = 0.329 and *p* = 0.407) after the 12-week intervention in either the control or walking group. However, between-group comparisons revealed significantly higher SOD activity (95% CI: 0.69 to 11.63, partial η^2^ = 0.068, *p* = 0.028) and significantly lower MDA levels (95% CI: 0.05 to 1.42, partial η^2^ = 0.067, *p* = 0.037) in the walking group compared with the control group (Figure 4).

### 3.4. Blood Pressure

Following the 12-week intervention, SBP (*p* < 0.001), DBP (*p* = 0.023), PP (*p* = 0.002), and MAP (*p* < 0.001) significantly decreased in the control group. However, PR significantly increased (*p* = 0.023), while the RPP showed no significant change (*p* = 0.517). In contrast, the walking group exhibited significant reductions in SBP, DBP, MAP, and RPP (all *p* < 0.001), whereas PR (*p* = 0.542) and PP (*p* = 0.117) remained unchanged (Table 3).

**Figure 5:**
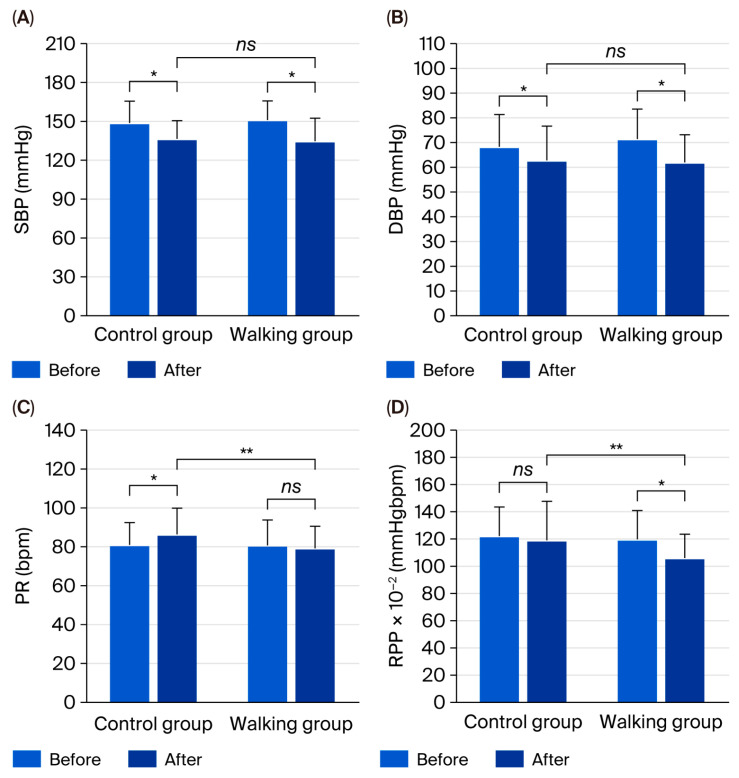
Systolic blood pressure (SBP) (**A**), diastolic blood pressure (DBP) (**B**), pulse rate (PR) (**C**), and rate–pressure product (RPP) (**D**) in control and walking groups at baseline and after 12 weeks. Data are mean ± SD. * *p* < 0.05 vs. before intervention; ** *p* < 0.05 vs. control group; *ns* = not significant (*p* > 0.05).

Between-group comparisons revealed significantly lower PR (95% CI = 1.33 to 12.35, partial η^2^ = 0.098, *p* = 0.016) and RPP (95% CI = 0.64 to 23.39, partial η^2^ = 0.073, *p* = 0.039) in the walking group compared with the control group (Figure 5). However, no significant differences were observed in SBP (95% CI = −6.49 to 8.15, partial η^2^ = 0.001, *p* = 0.822), DBP (95% CI = −2.46 to 7.90, partial η^2^ = 0.018, *p* = 0.298), PP (95% CI = −8.14 to 4.58, partial η^2^ = 0.005, *p* = 0.578), or MAP (95% CI = −3.35 to 7.06, partial η^2^ = 0.008, *p* = 0.478) between groups.

### 3.5. Body Composition and Fat Distribution

Body composition parameters, including BM (*p* = 0.412 and *p* = 0.350), BMI (*p* = 0.971 and *p* = 0.423), fat mass (*p* = 0.468 and *p* = 0.472), muscle mass (*p* = 0.559 and *p* = 0.611), bone mass (*p* = 0.333 and *p* = 0.730), VFL (*p* = 0.265 and *p* = 0.515), and BMR (*p* = 0.410 and *p* = 0.516), did not significantly change in either the control or walking group. Similarly, waist circumference (*p* = 0.332 and *p* = 0.052), hip circumference (*p* = 0.888 and *p* = 0.875), and the WHR (*p* = 0.178 and *p* = 0.058) showed no significant within-group changes, although a trend toward decreased waist circumference and WHR was observed in the walking group. No significant between-group differences were detected for any of these parameters (all *p* > 0.05) (Table 4).

### 3.6. Physical Fitness

Handgrip strength in the left hand (*p* = 0.467 and *p* = 0.263) showed no significant changes from baseline in either the control or walking groups. However, handgrip strength in the right hand significantly increased in the walking group (*p* = 0.038), whereas there was no significant alteration in the control group (*p* = 0.153). In addition, performance in the 60STS significantly increased in the walking group (*p* = 0.035), while no significant change was observed in the control group (*p* = 0.877). Post-intervention, the 60STS value was significantly higher in the walking group compared with the control group (*p* = 0.043). No significant between-group differences were found in left (*p* = 0.446) or right (*p* = 0.812) handgrip strength (Table 5).

### 3.7. Quality of Life

Following the intervention, the total QOL scores were categorized as good in both groups. Within-group analysis showed that the overall QOL score did not significantly change in the control group (*p* = 0.325), whereas it significantly increased in the walking group (*p* < 0.001). Furthermore, the post-intervention QOL score was significantly higher in the walking group compared with the control group (*p* < 0.001) (Table 5).

## 4. Discussion

At baseline, participants in both groups exhibited comparable clinical characteristics, typical of community-dwelling adults with early-stage CKD. Most were classified as stage 2 CKD, with preserved renal function, and a smaller proportion as stage 3. The similar distribution of renal stages, comorbidities, and behavioral characteristics between groups confirmed appropriate randomization and supported the validity of subsequent comparisons. These characteristics reflect that CKD frequently coexists with metabolic and lifestyle-related risk factors, highlighting the need for multifaceted management approaches.

After 12 weeks, renal function parameters—including serum creatinine, eGFR, and eCrCl—showed no significant within- or between-group differences. Previous evidence suggests that moderate-intensity exercise may help preserve kidney function by enhancing renal perfusion, improving endothelial function, and maintaining hemodynamic stability without imposing excessive physiological stress on the kidneys [24,25]. The absence of statistically significant changes in this study may be explained by the relatively short intervention duration and the predominance of participants with mild renal impairment, in whom kidney function typically remains stable over short observation periods [26].

Biochemically, the 12-week rhythmic walking intervention led to significant reductions in TC, LDLC, TG, and the TG/HDLC ratio, with a favorable trend in the TC/HDLC ratio. These results indicate improved lipid metabolism, possibly through enhanced lipoprotein lipase activity, greater fatty acid oxidation, and increased clearance of TG-rich lipoproteins [27,28]. Exercise may also promote reverse cholesterol transport by activating PPAR-α signaling and apolipoprotein A1 expression, thereby lowering atherogenic lipids [29]. The significant between-group differences in TC, TG, and TG/HDLC ratio confirm that rhythmic walking offers measurable cardiometabolic benefits beyond standard care. Considering that dyslipidemia contributes to both cardiovascular disease and renal deterioration via lipid accumulation, oxidative stress, and inflammation [30], the observed lipid-lowering effects are clinically meaningful and may confer long-term protection if maintained.

No significant changes were detected in BG, uric acid, or HDLC levels, suggesting that the exercise intensity and duration may have been insufficient to produce substantial improvements in glycemic control or purine metabolism. Previous studies indicate that longer or more vigorous interventions are typically required to reduce BG and uric acid concentrations [31,32]. Nonetheless, the absence of adverse changes supports the safety of rhythmic walking in individuals with mild-to-moderate CKD.

Regarding oxidative stress, no significant within-group changes in SOD activity or MDA levels were detected; however, between-group comparisons revealed significantly higher SOD activity and lower MDA levels in the walking group. These outcomes suggest a modest yet meaningful antioxidant effect, characterized by enhanced enzymatic defense against reactive oxygen species (ROS) and reduced lipid peroxidation. Regular aerobic exercise is known to improve redox homeostasis through the upregulation of key antioxidant enzymes—such as SOD, catalase, and glutathione peroxidase—and by decreasing mitochondrial ROS production [33,34]. The observed increase in SOD activity reflects an adaptive enhancement of endogenous antioxidant capacity in response to repeated exercise stimuli, thereby maintaining oxidative balance and protecting cellular structures from oxidative damage [35]. Conversely, the control group demonstrated a numerical rise in MDA levels (+8.98%), indicating a trend toward greater oxidative stress in the absence of regular physical activity. As oxidative stress is a hallmark of CKD and contributes to systemic inflammation and cardiovascular complications [36,37], these findings highlight the therapeutic relevance of exercise in this population. Mechanistically, the reduction in lipid peroxidation may be attributed to improved nitric oxide (NO) bioavailability, enhanced mitochondrial efficiency, and the downregulation of pro-oxidant pathways, including NADPH oxidase activity [38,39].

The lack of significant within-group effects may stem from the short intervention period, mild disease severity, and variability in diet, medication, and baseline redox status. In early-stage CKD, oxidative stress levels are relatively low, making measurable improvements more subtle over brief durations [40]. Nonetheless, the significant between-group differences highlight rhythmic walking as a viable nonpharmacological strategy to strengthen antioxidant capacity.

Both groups exhibited reductions in hemodynamic parameters after 12 weeks; however, the magnitude and pattern of improvement differed. The control group showed significant reductions in SBP, DBP, PP, and MAP. These improvements may be partly explained by the Hawthorne effect, wherein participants modify their behavior simply because they are aware of being observed, leading to temporary enhancements in health outcomes. Additionally, the standard care effect may have contributed, as participants in the control group continued to receive routine medical advice, pharmacological treatment, and lifestyle counseling from healthcare providers, all of which could have influenced BP regulation.

In contrast, the walking group demonstrated significant decreases in SBP, DBP, MAP, and RPP without an accompanying increase in PR, suggesting favorable cardiovascular adaptations to rhythmic walking. When comparing between groups, the walking intervention produced greater improvements in PR and RPP, underscoring its additional cardiovascular benefits, including enhanced cardiac efficiency and improved autonomic regulation, beyond those achieved through standard care alone. Furthermore, the reduction in RPP—an index of myocardial oxygen consumption—indicates a lower cardiac workload and improved cardiovascular performance, consistent with previous evidence of exercise-induced cardioprotection in CKD [41,42,43].

Low-intensity walking may reduce BP through enhanced endothelial NO bioavailability, reduced sympathetic tone, and improved baroreceptor sensitivity [44,45]. Exercise-induced shear stress promotes endothelial NO synthase expression, leading to vasodilation and reduced peripheral resistance [46]. Enhanced vascular compliance and decreased arterial stiffness likely explain the observed reductions in MAP [47]. Although between-group differences in SBP and DBP did not reach statistical significance, this may be due to well-controlled baseline BP and concurrent antihypertensive therapy. Exercise-induced BP reductions are typically greater in individuals with higher baseline values [48]. Moreover, the 12-week, low-intensity protocol may not have been sufficient to produce large between-group effects, despite clear trends favoring the walking group (−10.68% and −13.19% vs. −8.23% and −7.94% for SBP and DBP, respectively; reductions of 16.11 and 9.42 mmHg vs. 12.23 and 5.42 mmHg). The increase in PR in the control group may indicate autonomic imbalance, whereas the stable PR and reduced RPP in the walking group suggest improved vagal tone and cardiovascular efficiency [49].

Mechanistically, rhythmic walking may enhance lipid metabolism by increasing skeletal muscle lipoprotein lipase activity and improving peripheral insulin sensitivity, thereby facilitating TG clearance and reducing TC levels. In addition, repeated bouts of aerobic muscle activity help maintain redox balance through the upregulation of endogenous antioxidant defenses (e.g., SOD) and the attenuation of lipid peroxidation, as reflected by lower MDA levels. Improved endothelial function and increased NO bioavailability may further contribute to reductions in vascular resistance and BP. Collectively, these adaptive responses align with the favorable trends observed in lipid profile, oxidative stress markers, and hemodynamic parameters following the 12-week rhythmic walking intervention.

Body composition and anthropometric parameters—including BM, BMI, fat mass, muscle mass, bone mass, VFL, and BMR—remained unchanged after 12 weeks in both groups. Similarly, waist and hip circumferences and WHR did not change significantly, although a modest downward trend was observed in the walking group. These findings imply that the exercise program did not markedly affect overall body composition within the study period. The lack of change likely reflects the low exercise intensity, short duration, and absence of dietary modification. Significant reductions in fat mass typically require longer or higher-intensity programs [50,51]. Moreover, the older age of participants and the presence of CKD likely limited their metabolic adaptability and anabolic responses to low-intensity exercise [52,53].

Functional fitness improved significantly in the walking group, as evidenced by higher right-hand grip strength and greater repetitions in the 60STS test, whereas the control group showed no improvement. These results indicate enhanced muscular endurance and functional mobility, likely due to repeated muscle activation promoting mitochondrial biogenesis and improved oxygen utilization [54,55]. The 60STS test, a sensitive measure of lower-limb endurance and functional capacity, reflects meaningful gains in mobility that are crucial for maintaining independence and QOL in older adults with CKD [56,57].

After 12 weeks, QOL scores were classified as good in both groups, but significant improvement was observed only in the walking group. The control group showed no meaningful change, while post-intervention comparisons revealed higher QOL scores among participants who exercised. This demonstrates that rhythmic walking improved perceived well-being and daily functioning. Physical activity is a key determinant of health-related QOL in CKD, influencing both physical and psychosocial dimensions [58,59]. The observed improvements likely result from enhanced cardiovascular efficiency, muscular endurance, and metabolic regulation, translating into reduced fatigue and improved functional ability [60,61].

Beyond physiological benefits, exercise provides psychological and emotional advantages. Regular walking can alleviate anxiety, depression, and stress by stimulating endorphin release and neuroplasticity in mood-regulating brain regions [62]. In CKD populations, where psychological distress and treatment-related fatigue are common, such benefits contribute substantially to emotional well-being [63,64]. The rhythmic, structured nature of walking may also foster mindfulness, relaxation, and a sense of accomplishment.

The absence of QOL improvement in the control group suggests that standard care alone may not meaningfully affect perceived well-being in the short term. Conversely, even low-intensity walking can yield significant psychosocial gains, consistent with prior findings that low-impact aerobic exercise enhances self-efficacy, social engagement, and emotional stability in CKD [65,66]. The relatively large effect size (partial η^2^ = 0.253) observed in this study highlights the clinical relevance of these improvements beyond their statistical significance. Nevertheless, the magnitude of QOL change did not reach the minimal clinically important difference (MCID) previously reported for CKD populations, estimated at approximately 3–5 points for the KDQOL-36 or SF-36 summary scores [67,68]. Thus, while the improvement indicates a favorable trend toward enhanced perceived well-being, it should be interpreted as modest in clinical terms.

Although the between-group differences in study outcomes were relatively modest, these changes may still carry biological relevance. In individuals with CKD, even small improvements in oxidative balance, inflammatory status, and vascular regulation can contribute to renal preservation and cardiometabolic stability. The consistent trends observed across multiple parameters indicate a gradual physiological adaptation to rhythmic walking. Nonetheless, the potential impact of assay variability on the magnitude of these differences cannot be entirely ruled out. Minor fluctuations may have arisen from inherent assay sensitivity or inter-assay variation. To minimize analytical error, all biochemical measurements were conducted under standardized laboratory conditions using internationally validated protocols and stringent internal quality controls at both the hospital and RIA Laboratory Co., Ltd. Therefore, although the magnitude of change was limited, the direction of the observed effects remains physiologically plausible and clinically meaningful within the context of CKD management.

In summary, rhythmic walking represents an effective, low-cost, and accessible intervention that promotes comprehensive health benefits in individuals with CKD. The observed improvements reinforce the importance of incorporating structured physical activity into renal rehabilitation programs to address both physiological and psychosocial aspects of health. However, several limitations should be considered when interpreting the present findings. First, the final sample size was smaller than initially calculated. Although 74 participants were enrolled, data from only 64 were included in the final analysis due to withdrawal or unavailability for post-intervention assessments. This reduction in sample size may have decreased the statistical power and precision of the analysis, potentially contributing to borderline or non-significant results. Second, randomization was conducted according to the sequence of participant enrollment, following the principle of simple randomization. This method effectively minimized systematic differences between groups, as evidenced by the absence of significant differences in baseline clinical characteristics. However, the simplicity of the randomization and allocation procedures may have increased the potential risk of bias compared with more rigorous methods, such as computer-generated block randomization, stratified randomization, or the use of sealed opaque envelopes. Third, objective physical activity monitoring (e.g., pedometer or accelerometer) was not employed to quantify daily step counts and corroborate self-reported adherence. Exercise adherence was instead monitored using an exercise logbook and verified remotely through phone calls and the Line application. Fourth, the proposed mechanistic explanations are inferential, as the present findings are based on short-term biochemical changes rather than direct molecular assessments. Therefore, causal interpretations regarding long-term physiological adaptations should be made with caution. Finally, interpretation of QOL outcomes should also consider methodological limitations. The assessment of QOL relied on a self-reported instrument, which, although demonstrating good internal consistency and acceptable validity, may have been influenced by recall and social desirability bias. Moreover, potential observer or Hawthorne effects cannot be entirely excluded, as participants in the walking group may have been more motivated or attentive due to being observed, possibly inflating perceived improvements in QOL or related health outcomes.

Future studies with larger sample sizes are warranted to confirm these findings and strengthen the generalizability of the results. In addition, future research should assess the long-term sustainability of these effects and investigate combined interventions—such as walking integrated with nutritional counseling or mindfulness training—to further enhance clinical outcomes. Longitudinal trials of extended duration, involving larger cohorts and molecular-level assessments (e.g., inflammatory cytokines, endothelial function, and mitochondrial activity), are recommended to elucidate the long-term renal and cardiometabolic benefits of rhythmic walking. Additionally, integrating subjective and objective assessments of QOL—such as activity tracking, blinded evaluations, or mixed-methods designs—may improve the validity and interpretability of QOL outcomes in CKD intervention trials.

## 5. Conclusions

This randomized controlled trial demonstrated that a 12-week rhythmic walking program is a safe, feasible, and effective low-intensity exercise strategy for individuals with CKD stages 2–3. Although no significant changes were observed in serum creatinine, eGFR, or eCrCl within groups, the walking intervention produced meaningful physiological benefits, including enhanced antioxidant defenses—reflected by increased SOD activity and reduced MDA levels—and favorable hemodynamic adaptations, such as decreased PR and RPP.

These findings indicate that rhythmic walking effectively reduces oxidative stress and cardiovascular workload without adversely affecting renal function. The simplicity, accessibility, and low physical demand of rhythmic walking make it a practical and sustainable form of exercise for older adults and individuals with early-stage CKD, particularly in community and outpatient settings. Collectively, the present results provide clinically relevant evidence supporting rhythmic walking as a safe adjunct to renal rehabilitation programs aimed at improving oxidative balance and cardiovascular efficiency in routine practice.

## Figures and Tables

**Table 1:** Baseline clinical characteristics of participants.

Characteristics	Control Group (*n* = 33)	Walking Group (*n* = 31)
Age (years)	69.03 ± 6.96	66.45 ± 7.91
Sex (male/female) (*n*, %)	14 (42)/19 (58)	9 (29)/22 (71)
Stages of CKD		
CKD Stage 2 (*n*, %)	26 (78.79)	20 (64.52)
CKD Stage 3 (*n*, %)	7 (21.21)	11 (35.48)
Co-morbidities		
Hypertension (*n*, %)	31 (93.94)	29 (93.55)
Dyslipidemia (*n*, %)	13 (39.39)	18 (58.06)
Type 2 diabetes (*n*, %)	7 (21.21)	8 (25.81)
Old transient ischemic attack (*n*, %)	4 (12.12)	4 (12.90)
Gouty arthritis (*n*, %)	2 (6.06)	4 (12.90)
Thyroid disease (*n*, %)	2 (6.06)	0 (0)
Atrial fibrillation (*n*, %)	1 (3.03)	0 (0)
Asthma (*n*, %)	0 (0)	1 (3.23)
Urine protein		
Negative (%)	96.97	90.32
Current lifestyle factors		
Cigarette smoking (*n*, %)	6 (18.18)	1 (3.23)
Alcohol consumption (*n*, %)	8 (24.24)	6 (19.35)

Data are presented as mean ± SD, frequencies, and percentages.

**Table 2:** Kidney function and biochemical parameters of participants in the control and walking groups at baseline and after 12 weeks of intervention.

Parameters	Control Group (*n* = 33)			Walking Group (*n* = 31)			*p* Value (After vs. After)
	Before	After	Δ Change (%)	Before	After	Δ Change (%)	
Creatinine (mg/dL)	1.03 ± 0.22	1.02 ± 0.18	−0.01 (0.98)	1.05 ± 0.40	1.04 ± 0.24	−0.01 (0.95)	0.744
eGFR (mL/min/1.73 m^2^)	65.30 ± 10.05	65.89 ± 15.11	0.59 (0.90)	63.62 ± 12.81	66.83 ± 18.83	3.21 (5.05)	0.197
eCrCl (mL/min)	53.16 ± 18.57	54.01 ± 20.15	0.85 (1.60)	52.50 ± 13.92	55.50 ± 18.64	3.00 (5.71)	0.318
Uric acid (mg/dL)	5.92 ± 1.32	5.84 ± 1.37	−0.08 (1.37)	6.16 ± 1.64	6.08 ± 1.46	−0.08 (1.32)	0.896
Glucose (mg/dL)	112.36 ± 55.23	106.76 ± 38.50	−5.60 (5.25)	110.68 ± 43.14	102.97 ± 23.91	−7.71 (7.49)	0.633
TC (mg/dL)	172.45 ± 37.33	168.88 ± 50.94	−3.57 (2.07)	190.47 ± 33.59	164.90 ± 37.92 *,**	−25.57 (13.42)	0.030
HDLC (mg/dL)	48.30 ± 11.33	50.06 ± 11.84	1.76 (3.64)	47.29 ± 8.40	50.77 ± 15.96	3.48 (7.36)	0.490
LDLC (mg/dL)	117.13 ± 29.42	115.21 ± 40.72	−1.62 (1.38)	129.93 ± 38.20	112.10 ± 37.52 *	−17.83 (13.72)	0.086
TG (mg/dL)	133.19 ± 56.31	129.24 ± 56.36	−3.95 (2.97)	162.86 ± 63.93	130.15 ± 68.30 *,**	−32.71 (20.08)	0.024
TC/HDLC ratio	3.57 ± 0.86	3.55 ± 0.88	−0.02 (0.56)	4.12 ± 1.51	3.61 ± 1.08	−0.51 (12.38)	0.071
LDLC/HDLC ratio	2.48 ± 0.88	2.43 ± 0.76	−0.05 (2.06)	2.86 ± 1.32	2.49 ± 1.05	−0.37 (14.86)	0.170
TG/HDLC ratio	3.05 ± 2.46	2.86 ± 1.60	−0.19 (6.23)	3.88 ± 2.60	2.99 ± 1.49 *,**	−0.89 (22.94)	0.032

Data are presented as mean ± SD. eCrCl, estimated creatinine clearance; eGFR, estimated glomerular filtration rate; HDLC, high-density lipoprotein cholesterol; LDLC, low-density lipoprotein cholesterol; TC, total cholesterol; TG, triglyceride. * *p* < 0.05 vs. before intervention; ** *p* < 0.05 vs. control group.

**Table 3:** Blood pressure parameters of participants in the control and walking groups at baseline and after 12 weeks of intervention.

Parameters	Control Group (*n* = 33)			Walking Group (*n* = 31)			*p* Value (After vs. After)
	Before	After	Δ Change (%)	Before	After	Δ Change (%)	
PR (bpm)	81.12 ± 11.44	86.52 ± 13.53 *	5.40 (6.66)	80.90 ± 13.07	79.44 ± 11.24 **	−1.46 (1.80)	0.016
SBP (mmHg)	148.68 ± 16.78	136.45 ± 13.91 *	−12.23 (8.23)	150.85 ± 14.73	134.74 ± 17.67 *	−16.11 (10.68)	0.822
DBP (mmHg)	68.30 ± 13.06	62.88 ± 13.83 *	−5.42 (7.94)	71.42 ± 12.12	62.00 ± 11.18 *	−9.42 (13.19)	0.298
PP (mmHg)	82.55 ± 17.79	73.58 ± 13.58 *	−8.97 (10.87)	77.26 ± 17.37	72.74 ± 16.77	−4.52 (5.85)	0.578
MAP (mmHg)	95.82 ± 10.76	87.40 ± 12.29 *	−8.42 (8.79)	97.17 ± 11.17	86.25 ± 11.17 *	−10.92 (11.24)	0.478
RPP × 10^−2^ (mmHgbpm)	122.47 ± 21.29	119.26 ± 28.60	−3.21 (2.62)	119.87 ± 21.16	106.20 ± 17.48 *,**	−13.67 (11.40)	0.039

Data are presented as mean ± SD. DBP, diastolic blood pressure; MAP, mean arterial pressure; PP, pulse pressure; PR, pulse rate; RPP, rate–pressure product; SBP, systolic blood pressure. * *p* < 0.05 vs. before intervention; ** *p* < 0.05 vs. control group.

**Table 4:** Body composition and fat distribution parameters of participants in the control and walking groups at baseline and after 12 weeks of intervention.

Parameters	Control Group (*n* = 33)			Walking Group (*n* = 31)			*p* Value (After vs. After)
	Before	After	Δ Change (%)	Before	After	Δ Change (%)	
BM (kg)	57.82 ± 10.65	58.64 ± 10.93	0.82 (1.42)	59.21 ± 15.16	58.39 ± 15.68	−0.82 (1.38)	0.973
BMI (kg/m^2^)	24.87 ± 4.18	25.11 ± 3.98	0.24 (0.97)	24.43 ± 4.12	24.42 ± 4.43	−0.01 (0.04)	0.863
Fat mass (kg)	18.38 ± 8.51	18.87 ± 8.13	0.49 (2.67)	17.17 ± 8.58	17.53 ± 8.21	0.36 (2.10)	0.267
Muscle mass (kg)	39.55 ± 10.13	39.79 ± 10.06	0.24 (0.61)	37.30 ± 7.36	37.61 ± 7.29	0.31 (0.83)	0.928
Bone mass (kg)	2.14 ± 0.44	2.16 ± 0.42	0.02 (0.93)	2.21 ± 0.57	2.25 ± 0.57	0.04 (1.81)	0.638
Visceral fat level	8.94 ± 3.95	9.16 ± 3.66	0.22 (2.46)	10.18 ± 4.49	9.85 ± 4.70	−0.33 (3.24)	0.325
BMR (kcal)	1181.58 ± 286.31	1150.70 ± 351.34	−30.88 (2.61)	1098.26 ± 281.77	1128.68 ± 195.65	30.42 (2.77)	0.486
Waist circumference (cm)	86.81 ± 9.21	87.90 ± 8.95	1.09 (1.26)	88.28 ± 10.51	85.24 ± 10.79	−3.04 (3.44)	0.441
Hip circumference (cm)	96.30 ± 9.18	97.03 ± 8.77	0.73 (0.76)	96.74 ± 7.01	96.58 ± 7.05	−0.16 (0.17)	0.893
WHR	0.90 ± 0.06	0.91 ± 0.06	0.01 (1.11)	0.91 ± 0.06	0.88 ± 0.06	−0.03 (3.30)	0.487

Data are presented as mean ± SD. BM, body mass; BMI, body mass index; BMR, basal metabolic rate; WHR, waist-to-hip ratio.

**Table 5:** Physical fitness parameters and quality of life of participants in the control and walking groups at baseline and after 12 weeks of intervention.

Parameters	Control Group (*n* = 33)			Walking Group (*n* = 31)			*p* Value (After vs. After)
	Before	After	Δ Change (%)	Before	After	Δ Change (%)	
Left handgrip strength (kg)	20.28 ± 8.89	22.17 ± 15.24	1.89 (9.32)	19.21 ± 7.23	19.97 ± 6.85	0.76 (3.96)	0.446
Right handgrip strength (kg)	19.14 ± 9.15	20.32 ± 7.74	1.18 (6.17)	18.53 ± 8.24	20.24 ± 7.45 *	1.71 (9.23)	0.812
60STS (rep)	19.50 ± 4.08	20.35 ± 5.64	0.85 (4.36)	20.47 ± 6.96	22.67 ± 7.63 *,**	2.20 (10.75)	0.043
Total QOL score	103.61 ± 1.87	103.64 ± 1.85	0.03 (0.03)	103.23 ± 2.35	104.03 ± 2.24 *,**	0.80 (0.77)	<0.001

Data are presented as mean ± SD. 60STS, 60 s sit-to-stand; QOL, quality of life. * *p* < 0.05 vs. before intervention; ** *p* < 0.05 vs. control group.

## Data Availability

The data are available upon request from the corresponding author. Restrictions apply to the availability of these data due to privacy and ethical considerations.

## Abbreviations

The following abbreviations are used in this manuscript:

60STS	60 s sit-to-stand
ANCOVA	Analysis of covariance
ANOVA	Analysis of variance
BG	Blood glucose
BM	Body mass
BMI	Body mass index
BMR	Basal metabolic rate
BP	Blood pressure
CKD	Chronic kidney disease
DBP	Diastolic blood pressure
DNA	Deoxyribonucleic acid
eCrCl	Estimated creatinine clearance
eGFR	Estimated glomerular filtration rate
HDLC	High-density lipoprotein-cholesterol
LDLC	Low-density lipoprotein-cholesterol
MAP	Mean arterial pressure
MCID	Minimal clinically important difference
MDA	Malondialdehyde
NADPH	Nicotinamide adenine dinucleotide phosphate
NO	Nitric oxide
PP	Pulse pressure
PR	Pulse rate
QOL	Quality of life
ROS	Reactive oxygen species
RPP	Rate–pressure product
SBP	Systolic blood pressure
SD	Standard deviation
SOD	Superoxide dismutase
TC	Total cholesterol
TG	Triglyceride
VFL	Visceral fat level
WHO	World Health Organization
WHR	Waist-to-hip ratio
